# Metacognitive deficits are associated with lower sensitivity to preference reversals in nicotine dependence

**DOI:** 10.1038/s41598-022-24332-0

**Published:** 2022-11-17

**Authors:** Alexander Soutschek, Adam Bulley, Charlotte E. Wittekind

**Affiliations:** 1 Chair of Experimental and General Psychology, Department of Psychology, Ludwig Maximilian University Munich, Leopoldstr. 13, 80802 Munich, Germany; 2 The University of Sydney, School of Psychology and Brain and Mind Centre, Sydney, Australia; 3 Department of Psychology, Harvard University, Cambridge, USA; 4 Chair of Clinical Psychology, Department of Psychology, Ludwig Maximilian University Munich, Munich, Germany

**Keywords:** Human behaviour, Addiction

## Abstract

Deficits in impulse control belong to the core profile of nicotine dependence. Smokers might thus benefit from voluntarily self-restricting their access to the immediate temptation of nicotine products (precommitment) in order to avoid impulse control failures. However, little is known about how smokers’ willingness to engage in voluntary self-restrictions is determined by metacognitive insight into their general preferences for immediate over delayed rewards. Here, with a series of monetary intertemporal choice tasks, we provide empirical evidence for reduced metacognitive accuracy in smokers relative to non-smokers and show that smokers overestimate the subjective value of delayed rewards relative to their revealed preferences. In line with the metacognitive deficits, smokers were also less sensitive to the risk of preference reversals when deciding whether or not to restrict their access to short-term financial rewards. Taken together, the current findings suggest that deficits not only in impulse control but also in metacognition may hamper smokers’ resistance to immediate rewards and capacity to pursue long-term goals.

## Introduction

Tobacco smoking is a major risk factor for several chronic diseases including cancer, lung diseases, and cardiovascular diseases^1^, imposing immense costs to individuals and society. A crucial question is why smokers so often fail in smoking cessation despite wanting to quit and knowing the potentially deadly long-term consequences. The high rate of relapse in chronic nicotine dependence is thought to at least partially stem from deficits to impulse control and exaggerations in the steepness of delay discounting^2–4^, alongside various cognitive and social factors^5,6^. Delay discounting refers to the decline in the subjective value of rewards with time until their receipt, as seen for instance when individuals prefer an immediate reward (e.g., smoking a cigarette) over a long-term outcome (e.g., avoidance of negative long-term consequences of smoking)^7^. A large body of evidence suggests that smokers devalue outcomes in the future more strongly than non-smokers (for a meta-analysis, see^8^), with steeper discounting of future outcomes predicting both a higher risk of smoking initiation and lower rates of successful quitting^9^.

Despite evidence for impulse control deficits and steeper delay discounting in nicotine dependence, smokers often deny having problems with delay discounting and overestimate their capacity to quit smoking^10–12^. A discrepancy between objective smoking-related decisions and self-reported beliefs about those decisions might indicate deficits in metacognitive accuracy. The term “metacognitive accuracy” (or “metacognitive sensitivity”) refers to the ability to monitor, evaluate, and reliably report the accuracy of decision processes^13,14^. Recent theoretical accounts highlight the moderating role of metacognitive beliefs for substance use behavior^15–17^. For example, refusal self-efficacy—confidence in the ability to refuse smoking—moderates smoking behavior in adolescents^18,19^. However, these accounts mainly focus on the contents of metacognitive beliefs (e.g., beliefs about the controllability of one’s desires), whereas the role of the accuracy of metacognitive beliefs for substance use behavior remains unknown. Thus, despite the evidence that smokers might be too optimistic regarding their impulse-control capacities or preferences for delayed over immediate rewards, it has never been experimentally tested whether the accuracy of metacognitive processes is lower in smokers than in non-smokers.

Understanding whether smokers show not only differences in impulse control or delay discounting but also in metacognition is important given that not only objective time preferences but also subjective beliefs about one’s preference for future over immediate rewards guide human behavior^7,20–22^. For example, only if smokers anticipate that they will give in to smoking a cigarette when going to a party can they avoid such situations where their capacity to resist immediate temptations would not be sufficient. Conceptually, prospective decisions where decision makers voluntarily restrict their access to temptations presuppose metacognitive awareness of one’s preferences for delayed versus immediate rewards^7,21,23^. Recent findings suggest that better metacognitive accuracy indeed predicts a higher likelihood of restricting one's access to immediate rewards when anticipating potential preference reversals^24,25^. If smokers overestimate their preferences for future over immediate rewards due to lack of metacognitive insight into their preferences, they may thus also show a lower willingness to precommit to long-term rewards in order to avoid preference reversals (i.e., switching from one’s current preference for the delayed reward to preferring the smaller-sooner reward when re-deciding in the future). From this perspective, metacognitive awareness of one’s preferences for delayed versus immediate rewards represents the precondition for the ability to accurately anticipate potential preference reversals from delayed to immediate rewards. As the anticipation of potential preference reversals belongs to the driving forces of precommitment decisions^7,23^, deficits in metacognition may thus reduce the sensitivity to preference reversals during precommitment decisions. Metacognitive deficits in nicotine addiction might also challenge the theory of rational addiction according to which substance abuse can be understood as rational and farsighted maximization of an individual’s utility^26,27^. Conceptualizing addiction as rational forward-looking behavior presupposes that decision makers possess accurate representations of their economic preferences. Evidence for metacognitive deficits in addiction would therefore challenge the plausibility of this account. However, because research on delay discounting in smokers (and other forms of substance abuse/dependence in general) has mainly focused on the objective tendency to choose delayed over immediate rewards, it remains unknown whether smokers indeed have a reduced willingness to precommit to avoid preference reversals compared to non-smokers, and how any such changes might be related to possible metacognitive deficits.

**Figure 1:**
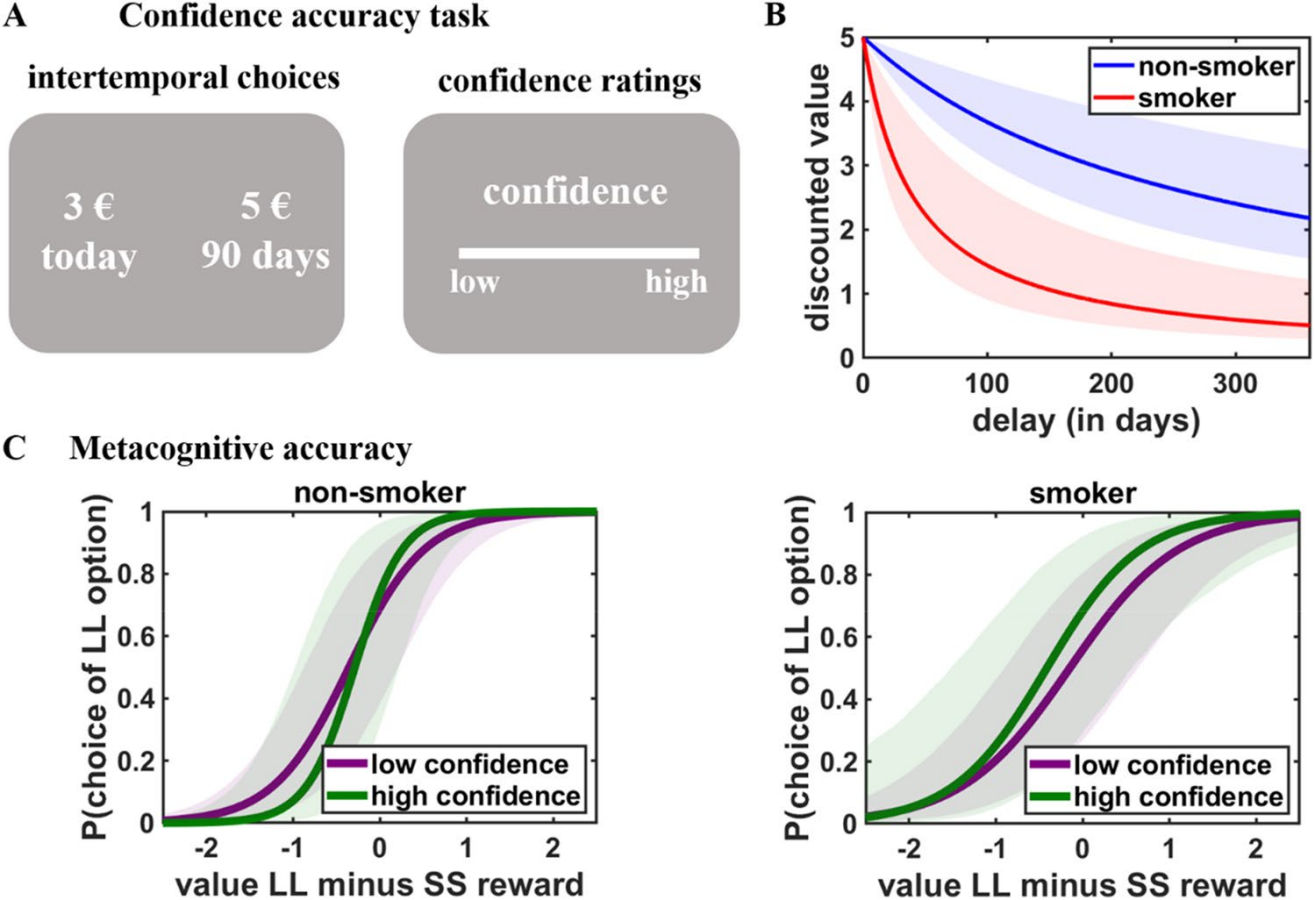
(**A**) In the confidence accuracy task, participants made choices between smaller-sooner (SS; e.g., 3 euro today) and larger-later (LL; e.g., 5 euro in 90 days) rewards. After each choice, they had to rate their subjective confidence to have made the best possible choice. (**B**) Consistent with previous findings, smokers discounted future rewards steeper than non-smokers. (**C**) Smokers also showed lower metacognitive accuracy than non-smokers, as indicated by a smaller difference between the slopes (which capture decision uncertainty) for low versus high confidence. For illustration purpose, we split confidence ratings into low versus high confidence trials. Shaded areas indicate 95% confidence intervals.

Here, we addressed these questions by investigating whether smokers, relative to non-smokers, show metacognitive deficits and a lower demand for precommitment (i.e., binding, irreversible choices of delayed rewards). First, we hypothesize that smokers, compared with non-smokers, have worse metacognitive accuracy regarding their preferences for delayed over immediate outcomes and more strongly overestimate the value of future rewards relative to their revealed preferences (i.e., preferences for delayed versus immediate rewards as determined via observable choice behavior rather than self-report). Second, we expect that smokers, relative to non-smokers, also show a lower sensitivity to the risk of potential preference reversals when making decisions about whether or not to precommit to long-term rewards, with the metacognitive deficits (see first hypothesis) statistically explaining the lower sensitivity to preference reversals. To test these hypotheses, we administered decision tasks that allow quantifying the degree of metacognitive awareness of one’s preferences for delayed over immediate rewards. In the confidence accuracy task, 37 smokers and 38 non-smokers made choices between smaller-sooner (SS; e.g., 3 euro today) and larger-later (LL; e.g., 5 euro in 90 days) monetary rewards (Fig. 1A). After each choice, they rated their subjective confidence in having made the best possible choice. In line with previous procedures^24,25,28^, this allows determining the degree to which individuals can reliably report uncertainty in the choice process while deciding between SS and LL rewards (“metacognitive accuracy”). Moreover, we asked participants to estimate the subjective value of monetary rewards in the future, and compared these self-reported estimates with the subjective values derived from choice behavior^28^. If smokers overestimate the values of future rewards relative to their revealed preferences, self-reported values of future rewards should be higher than the observed reward values determined by participants’ choices in the decision task. Lastly, to assess how metacognitive insight influences the willingness to restrict access to short-term rewards, participants also performed a precommitment task where they decided between making binding choices for LL rewards and postponing the final decision, which bears the risk that they might reverse their preference for the LL to the SS reward when having to make the final decision^24,25^. If smokers have less insight into how their preferences change over time and overestimate the value of future rewards, we expect them to be less sensitive to the risk of potential preference reversals in the precommitment task than non-smokers.

## Results

First, we aimed to replicate previous findings of steeper delay discounting in nicotine dependence. We compared the posterior distributions of the group-level hyperbolic discount parameters between smokers and non-smokers by subtracting the posterior distribution of the discount factor in the smoker group from the posterior distribution in the nonsmoker group (if the 95% HDI of the differences between the group-specific posterior distributions does not contain zero, the group difference can be considered as being significant). Consistent with the literature^8^, this comparison revealed that the discount factor k was significantly larger (indicating steeper delay discounting) in the smoker than in the non-smoker group, HDI_mean_ = -0.023, HDI_95%_ = [− 0.042; − 0.005] (Fig. 1B). Smokers and non-smokers were comparable with respect to age, sex, educational status, working memory capacity, and verbal intelligence, all *p*s > 0.27 (Table 1), suggesting that the difference in delay discounting cannot be explained by any pre-existing differences in these variables. However, there were significant group differences in the BIS and BAS scales, both *p* < 0.05, consistent with previous evidence^29^. Note that all statistical analyses were robust to adding the BIS/BAS scores as well as age and educational status as covariates of no interest.

**Table 1:** Demographic characteristics of the non-smoker and smoker groups.

Variable	Non-smoker	Smoker	*p*
Age	27.1 (8.5)	29.3 (9.6)	0.29
Sex	24 female/14 male	23 female/13 male	0.95
Years of education	14.2 (1.6)	14.1 (1.6)	0.73
Audit-C	2.7 (1.7)	3.1 (1.6)	0.27
BIS	3.2 (0.5)	2.8 (0.6)	0.003
BAS	3.2 (0.2)	3.1 (0.3)	0.05
MWTB	28.9 (3.2)	28.1 (3.7)	0.26
Digit span backward	2.8 (1.5)	2.3 (1.8)	0.18
Cigarettes/day		13.9 (4.1)	
Fagerström test		4.3 (1.6)	
%LL choices	69% (19)	53% (24)	0.003
Reported confidence	8.1 (0.9)	7.8 (0.9)	0.21
Value bias	0.3 (1.1)	1.1 (1.9)	< 0.001
%precommitment	32 (29)	37 (25)	0.46

We report group-specific means, standard deviations are in brackets. We also report the percentage of larger-later (LL) choices and mean confidence ratings in the confidence accuracy task, the mean value bias (self-reported minus revealed LL reward values) in the bidding task, and the percentage of precommitment choices in the precommitment task.

We next asked whether smokers and non-smokers also differ in their metacognitive accuracy. Following previous procedures^24,25^, we assessed metacognitive accuracy with a mixed generalized linear model (MGLM) where binary choices in the confidence accuracy task were regressed on predictors for Group (non-smoker versus smoker), difference in value (DV; value LL reward minus value SS reward), confidence, and the interaction terms. The strength of the interaction between DV (as measure of objective decision uncertainty) and confidence (subjective uncertainty) is an indicator of metacognitive accuracy. This is because participants should be more confident that they made the best choice the larger the difference in subjective value between the options, and the DV × Confidence interaction measures participants’ ability to track and report the consistency of their decisions. Smokers made significantly fewer LL choices than non-smokers, HDI_mean_ = − 3.33, HDI_95%_ = [− 5.53; − 1.35]. Moreover, the main effects of both DV, HDI_mean_ = 5.76, HDI_95%_ = [4.87; 6.76], and confidence, HDI_mean_ = 1.08, HDI_95%_ = [0.64; 1.57], were significant, the latter suggesting that non-smokers reported higher confidence after LL relative to SS choices. This association between higher confidence and LL choices was significantly reduced in the smoker group, Group × Confidence: HDI_mean_ = − 1.00, HDI_95%_ = [− 1.64; − 0.45], and smokers also showed lower choice consistency (as the predictor DV indicates how consistently participants’ choices are guided by the strength of their preference for one option over the other), Group × DV: HDI_mean_ = − 1.68, HDI_95%_ = [− 2.93; − 0.55]. The DV × Confidence interaction, HDI_mean_ = 1.85, HDI_95%_ = [1.38; 2.34], suggested that non-smokers could reliably track and report uncertainty in decision making, a measure of metacognitive accuracy. Importantly, metacognitive accuracy was significantly reduced in smokers compared to non-smokers, HDI_mean_ = − 0.64, HDI_95%_ = [− 1.19; -0.08] (Fig. 1C and Table 2). The significant Group × DV × Confidence interaction was robust to adding BIS/BAS scores, age, and educational status as covariates of no interest, HDI_mean_ = − 0.59, HDI_95%_ = [− 1.15; − 0.04]. This suggests that, in addition to their steeper delay discounting, smokers possess worse metacognitive access to the accuracy of their intertemporal choice process.

**Table 2:** Results of Bayesian MGLM in the confidence accuracy task, regressing binary choices (0 = immediate reward, 1 = delayed reward) on predictors for Group (0 = nonsmoker, 1 = smoker), difference in value (DV), confidence, and the interaction terms.

Predictor	Mean	2.5%	97.5%
Intercept	4.17 (0.77)	2.70	5.75
Group	− 3.33 (1.05)	− 5.53	− 1.35
DV	5.76 (0.48)	4.87	6.76
Confidence	1.08 (0.24)	0.64	1.57
Group × DV	− 1.68 (0.59)	− 2.93	− 0.55
Group × Confidence	− 1.00 (0.30)	− 1.64	− 0.45
DV × Confidence	1.85 (0.24)	1.38	2.34
Group × DV × Confidence	− 0.64 (0.28)	− 1.19	− 0.08

We report the upper and lower borders of the 95% HDI of the posterior distributions. Standard errors of the mean are in brackets.

**Figure 2:**
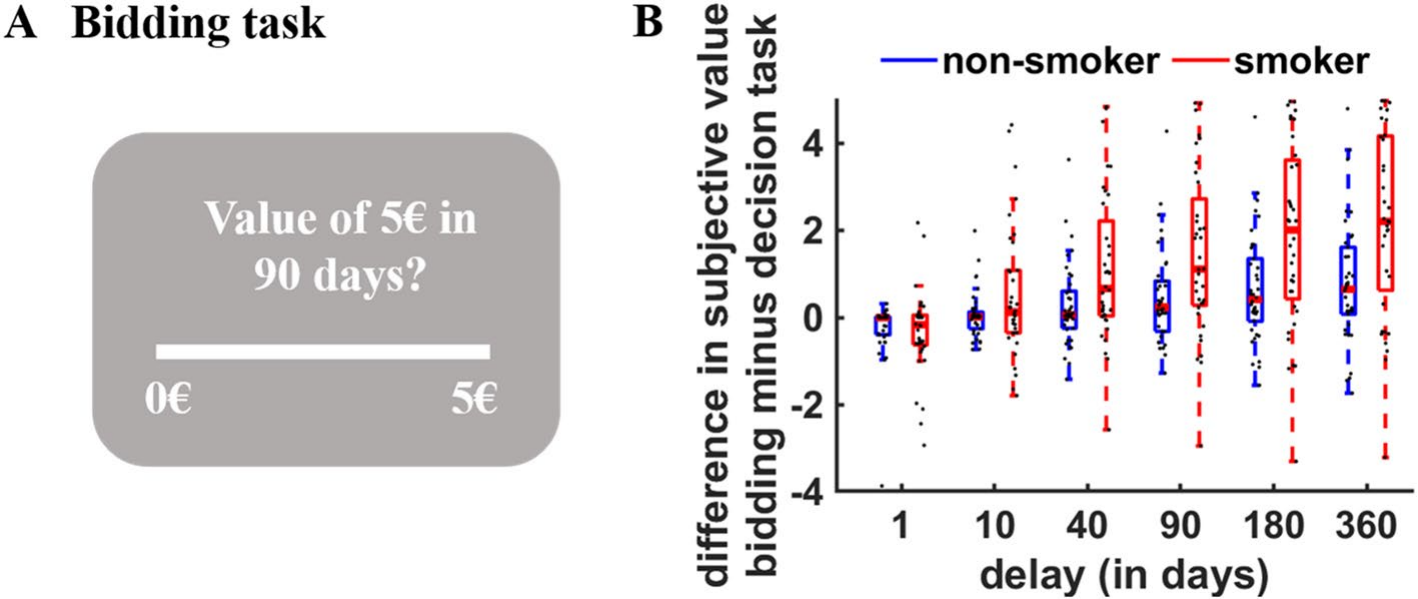
(**A**) In the bidding task, participants indicated which immediate reward magnitude they consider as equivalent to a given reward delivered in the future on a rating scale from 0 to 5 euro. The difference between subjective reward values estimated from observed choices in the confidence accuracy task (via individual hyperbolic discount parameters) and the self-reported values from the bidding task reflects the degree to which individuals over- or underestimate the value of future rewards relative to their revealed preferences. (**B**) Smokers, relative to non-smokers, overestimate their preferences for delayed over future rewards, particularly for longer delays, as indicated by higher self-reported than observed subjective values. Black dots indicate individual data points.

The observed deficits in metacognitive accuracy raise the question as to whether smokers are less able to recognise and correct for any biases in their intertemporal decision-making. Given that smokers show steeper delay discounting than non-smokers, it seems plausible to assume that they more strongly overestimate the value of future rewards relative to their revealed preferences. To test this, we compared the differences between self-reported subjective values of delayed rewards (bidding task) and the choice-based, revealed subjective values (confidence accuracy task) between study groups (value bias; Fig. 2A). In the control group, participants significantly overestimated the value of future rewards for longer delays, HDI_mean_ = 0.31, HDI_95%_ = [0.13; 0.49]. Crucially, smokers more strongly overestimated the value of future rewards than non-smokers, HDI_mean_ = 0.78, HDI_95%_ = [0.23; 1.34], and this group difference was stronger for longer delays, Group × Delay: HDI_mean_ = 0.39, HDI_95%_ = [0.13; 0.65] (Fig. 2B and Table 3). This suggests that smokers, compared with non-smokers, overestimate their preferences for delayed over immediate rewards. Again, the results of this analysis were robust to controlling for BIS/BAS scores, age, and educational status, HDI_mean_ = 0.40, HDI_95%_ = [0.13; 0.65]. The strength of this bias was negatively correlated with individual differences in metacognitive accuracy (individual coefficients for the DV × Confidence interaction), r = − 0.64, *p* < 0.001. This suggests that smokers’ overestimation of their preference for future over immediate rewards correlates with their deficits in metacognitive accuracy.

**Table 3:** Results of Bayesian MGLM for the bidding task, regressing differences between revealed and self-reported subjective values on predictors for Group (0 = nonsmoker, 1 = smoker), Delay, and the interaction term.

Predictor	Mean	2.5%	97.5%
Intercept	0.34 (0.20)	− 0.05	0.73
Group	0.78 (0.28)	0.23	1.34
Delay	0.31 (0.09)	0.13	0.49
Group × Delay	0.39 (0.13)	0.13	0.65

We report the upper and lower borders of the 95% HDI of the posterior distributions. Standard errors of the mean are in brackets.

**Figure 3:**
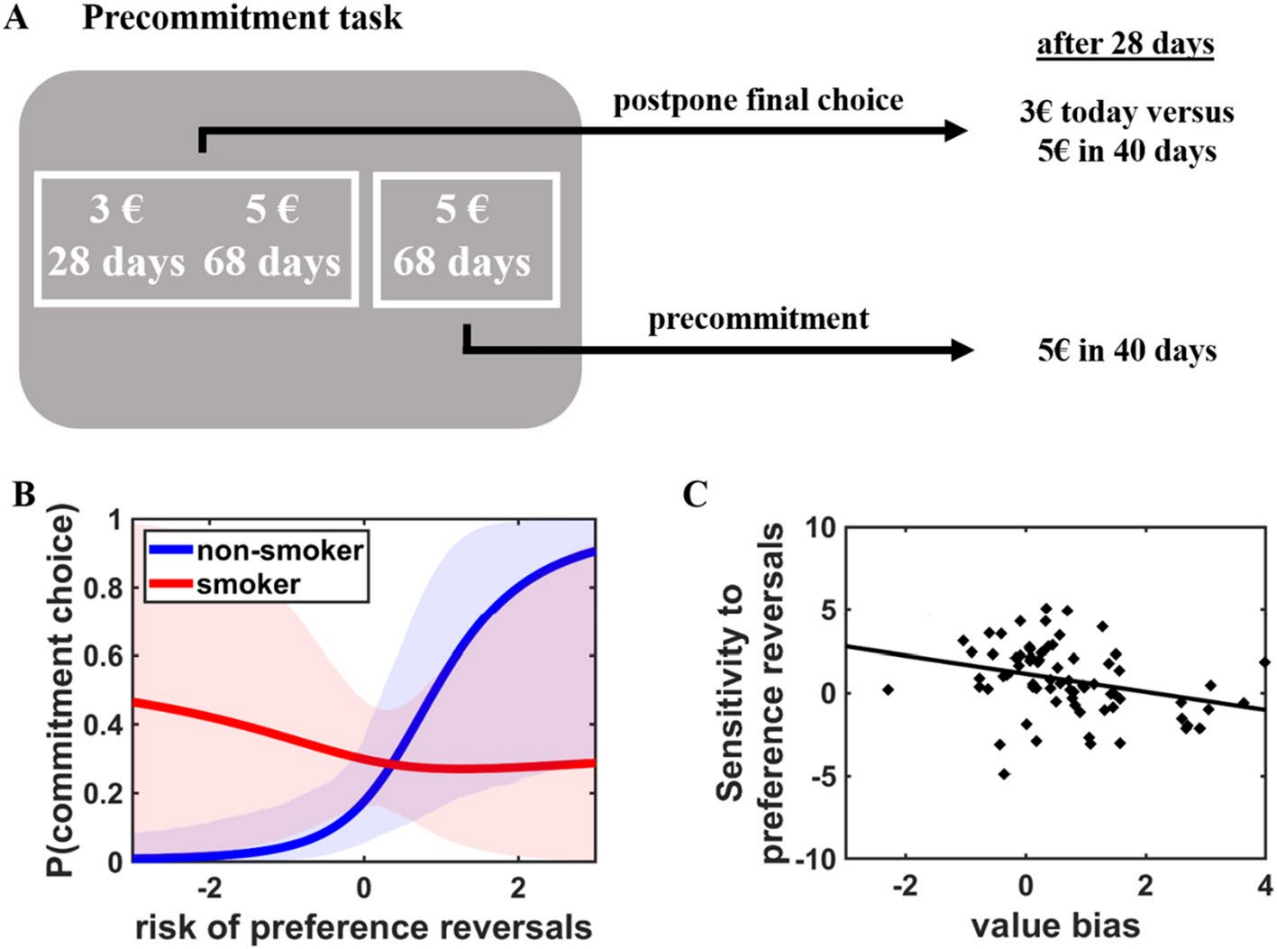
(**A**) In the precommitment task, participants opted between making a binding choice for a larger-later reward (e.g., 5 euro in 68 days) or postponing the decision. In the latter case, participants were re-contacted after 28 days and had to make a final choice between the adjusted reward options (in this example, “3 euro today versus 5 euro in 40 days”). (**B**) While non-smokers more strongly preferred to make a binding choice with increasing risk of preference reversals (i.e., the preference switches from the larger-later to the smaller-sooner option when being re-contacted after 28 days), smokers’ precommitment choices were unaffected by potential preference reversals. (**C**) The sensitivity to preference reversals correlated with individual differences in value bias, i.e. the degree to which individuals overestimate their preference for delayed over immediate rewards. Shaded areas in (**B**) indicate 95% confidence intervals.

Distorted representations of one’s time preferences may have negative consequences for prospective decision making, considering that individuals may prefer to restrict their access to immediate temptations particularly when anticipating that their impulse control will not be sufficient to resist the temptation^24^. We therefore assessed whether smokers, due to their metacognitive deficits, also show a lower willingness to precommit to long-term rewards. The precommitment task allowed us to assess the willingness to precommit as a function of the individual risk of preference reversals (Fig. 3A). Reversal risk was defined as the value difference between the LL and SS reward from participants’ current perspective minus the value difference between these options when participants had to make a final decision 28 days later. Higher values of this variable indicate that an individual is likely to switch from preferring the LL over the SS reward to preferring the SS over the LL reward when re-deciding between these options 28 days later. Smokers did not generally (i.e., irrespective of the risk of preference reversals) make fewer precommitment choices than non-smokers, HDI_mean_ = 0.69, HDI_95%_ = [− 0.62; 2.05]. However, while non-smokers increasingly preferred to precommit with a higher risk of preference reversals, HDI_mean_ = 1.85, HDI_95%_ = [0.03; 3.67], smokers were significantly less sensitive to the risk of preference reversals than non-smokers, Group × Reversal risk: HDI_mean_ = − 2.25, HDI_95%_ = [− 4.45; − 0.06] (Fig. 3B and Table 4). The Group × Reversal risk interaction remained significant when controlling for BIS/BAS scores, age, and educational level, HDI_mean_ = − 2.21, HDI_95%_ = [− 4.45; − 0.04]. A separate MGLM for the smoker group revealed no significant effect of Reversal risk on precommitment choices in smokers, HDI_mean_ = − 0.17, HDI_95%_ = [− 1.59; 1.26]. Thus, smokers are less willing to restrict their access to immediate rewards if the risk of preference reversals is high, perhaps because they underestimate their preferences for immediate over delayed rewards. In line with this interpretation, individual differences in the influence of the risk of preference reversals on precommitment choices (individual coefficients for Reversal risk) were significantly correlated with value bias (individual intercepts extracted from the MGLM in the bidding task), r = − 0.32, *p* = 0.006 (Fig. 3C), as well as with metacognitive accuracy (individual coefficients for DV × Confidence from the MGLM in the confidence accuracy task), r = 0.36, *p* = 0.002. Note that the correlation between Reversal risk and Value bias remained significant when using absolute rather than signed scores for Value bias, r = − 0.35, *p* = 0.003. This suggests that individuals with better metacognitive skills or who less overestimate their preference for LL over SS rewards (relative to their revealed preferences) also show a stronger sensitivity to potential preference reversals when making precommitment choices.

**Table 4:** Results of Bayesian MGLM in the precommitment task, regressing precommitment choices (0 = no precommitment, 1 = precommitment) on predictors for Group (0 = nonsmoker, 1 = smoker), difference in value (DV), reversal risk (DV in 28 days minus DV now), and the interaction terms.

Predictor	Mean	2.5%	97.5%
Intercept	− 1.69 (0.50)	− 2.65	− 0.69
Group	0.69 (0.69)	− 0.62	2.05
DV	− 0.43 (0.99)	− 2.40	1.52
Reversal risk	1.85 (0.93)	0.03	3.67
Group × DV	− 1.90 (1.23)	− 4.33	0.42
Group × Reversal risk	− 2.25 (1.13)	− 4.45	− 0.06
DV × Reversal risk	0.14 (0.14)	− 0.12	0.41
Group × DV × Reversal risk	− 0.04 (0.16)	− 0.36	0.29

We report the upper and lower borders of the 95% HDI of the posterior distributions. Standard errors of the mean are in brackets.

Next, we tested whether smokers’ value bias statistically explains the weaker influence of future preference reversals on precommitment choices with a mediation analysis. For this purpose, we re-computed the MGLM for the precommitment task and added the individual coefficients for value bias as predictor. This allowed us to assess whether the significant effect of smoking status on the sensitivity to reversal risk can be explained by the group differences in value bias (mediator variable). The result of a Sobel test assessing the significance of the indirect path (i.e., whether group differences in sensitivity to reversal risk are mediated by value bias), *t* = 2.02, *p* = 0.04, suggests that the degree to which individuals overestimate the value of LL rewards relative to their revealed preferences indeed mediates group differences in the sensitivity to Reversal risk. When we assessed whether smokers’ lower sensitivity to preference reversals is statistically mediated by the group difference in metacognitive accuracy, the Sobel test showed only a marginally significant result, *t* = 1.94, *p* = 0.05. Taken together, this suggests that discrepancies between self-reported and revealed subjective values (at least partially) explain why smokers are worse in anticipating and acting upon potential preference reversals.

## Discussion

Delay discounting is elevated in nicotine dependence^8,9^ and predicts (alongside other factors) both smoking initiation and the likelihood of relapsing^2,3,30^. However, less is known about the role of smokers’ metacognitive awareness of their time preferences. Here, we provide evidence for metacognitive deficits in nicotine dependence and their influence on prospective decision making. Smokers, relative to non-smokers, could less reliably report decision uncertainty in intertemporal decision making (metacognitive accuracy). Smokers also overestimated the self-reported value of future rewards relative to their revealed preferences. The finding that smokers overestimate their preference for future over immediate rewards informs previous questionnaire data according to which smokers are rather optimistic regarding their capacity to quit smoking^10–12^. While previous research has not directly compared self-reported with revealed time preferences, we provide evidence that smokers, compared with non-smokers, overestimate their preference for future over immediate rewards. Thus, nicotine dependence is characterized not only by steeper delay discounting but also by weaker metacognitive insight into time preferences and a stronger discrepancy between self-reported and revealed preferences for delayed rewards. We note that this discrepancy should not be necessarily interpreted as reflecting a metacognitive deficit, as choosing between LL and SS rewards (confidence accuracy task) might require different processes than estimating the value of LL rewards (bidding task). For example, choosing LL over SS rewards is thought to require higher-order control processes^31^. Deficits in such control processes might therefore contribute to the larger discrepancy between self-reported and revealed subjective values in smokers compared to non-smokers.

Metacognitive deficits may contribute to deficient prospective decision making in nicotine dependence by impairing the anticipation of potential preference reversals. This interpretation is corroborated by the findings in the precommitment task where the risk of changing the preference from LL to SS rewards in future choices showed a weaker impact on smokers’ than on non-smokers’ precommitment decisions. Replicating previous findings^25^, metacognitive abilities significantly correlated with the individual sensitivity to preference reversals, which supports the assumption that the efficient use of precommitment requires metacognitive insight into one’s time preferences. The significant mediation analysis suggests that because smokers overestimate their preference for delayed over immediate rewards, they may have difficulties in anticipating future situations where they might give in to the temptation to smoke despite currently preferring not to smoke (though one should hesitate to infer causal links between metacognition and precommitment from the mediation analysis). A lower sensitivity to preference reversals might potentially increase the risk of relapses^32^, albeit this remains speculative and will need to be tested with more ecologically valid measures of preference reversals in nicotine addiction.

The current findings have important implications for our conceptual understanding of delay discounting and impulse control deficits in nicotine dependence. Substance dependence can be conceptualized as arising at least in part from a dysfunctional dominance of the desire for immediate rewards over the pursuit of long-term goals^33–35^. Correspondingly, previous research focused on delay discounting in intertemporal choice as a behavioral marker of the preference for immediate rewards in addiction^8,9^. Additionally pharmacological or brain stimulation-based treatments in substance dependence/abuse aim at re-calibrating the balance between brain mechanisms involved in impulse control and wanting^36–39^.

Besides impulse control, however, voluntary self-restrictions are also an effective strategy which improve resistance to immediate temptations^7,40^. Precommitment devices where individuals voluntarily self-restrict their access to the desired substance have been designed to improve pharmacological treatments of substance dependence^41,42^. However, the use of such precommitment devices presupposes that patients possess metacognitive access to their time preferences. The current findings suggest that this metacognitive insight might be impaired in individuals with substance use disorders, which may hamper the efficient use of such precommitment devices. An important implication of our findings is that therapeutic neural interventions should target not only brain regions involved in impulse but also in metacognitive processes, for example the frontopolar cortex^25,43,44^, as enhancing metacognition may optimize the use of precommitment strategies. This is supported by evidence for abnormal activation in anterior prefrontal cortex during performance monitoring processes in addiction^17^, which may form the neural substrate of the observed metacognitive deficits. Alternatively, behavioral interventions like metacognitive training^45^ may allow for a reduction of dysfunctional metacognitive biases in substance dependence.

The current findings may also shed a new light on economic theories of addiction. According to the influential theory of rational addiction, substance abuse can be conceptualized as rational behavior that maximizes an individual’s utility function^26,27^. However, if smokers possess reduced metacognitive insight into their economic time preferences (as evidenced by the current findings), it seems questionable whether they can indeed integrate all future benefits and costs of substance abuse, which challenges the assumptions of the theory of rational addiction.

It is worth mentioning some limitations of the current study: first, the administered experimental tasks used monetary rewards, whereas impulsive decisions in nicotine dependence require trading-off the immediate consumption of cigarettes versus the negative long-term consequences of smoking. Note that the use of a dependence-unrelated currency was necessary in order to compare behavior between smokers and non-smokers, and there is evidence that the proposed link between metacognition and precommitment holds also for primary rewards^24,43,46^. Moreover, smokers discount monetary rewards and cigarettes to a similar extent^47^. While we are therefore optimistic that the reported findings will hold also for intertemporal decisions involving substance-related rewards, this will need to tested by future studies employing more ecologically valid measures of impulsiveness and craving in addiction. We note that decisions may not only be motivated by the desire for immediate rewards but also by the desire for avoiding the aversive state of craving^48^, whereas the current task measured only the preference for immediate rewards. As further limitation, we note that smokers made choices in a “cold” state as we placed no restrictions on their smoking before the experiment. We are thus cautious in drawing any conclusions regarding smokers’ behavior in a “hot” craving state, in which smokers have been found to overestimate their desire for future cigarette consumption compared to when being in a “cold” state^49^. Moreover, we did not control for participants’ income or socio-economic status, although these factors might potentially contribute to the observed group differences in delay discounting. Finally, one should keep in mind that the delay used in the confidence accuracy task (with a maximum delay of one year) are relatively short-term compared with the long-term consequences of smoking, which further constrains the clinical relevance of our current findings.

Taken together, the current findings inform theoretical accounts of substance dependence by providing evidence that—besides elevated delay discounting and deficits in impulse control—nicotine dependence is characterized by metacognitive deficits. These metacognitive deficits, in turn, may hamper the identification of situations where smokers’ impulse control capacities are not sufficient to resist temptations.

## Materials and methods

### Participants

Thirty-seven regular smokers (23 females, mean_age_ = 29.3 years, range = 19–54) and 38 non-smokers participated in the study (24 females, mean_age_ = 27.1 years, range = 19–55). Participants were recruited via advertisement and the internal participant pools of the chair of experimental psychology as well as of the Munich Experimental Laboratory for Economic and Social Sciences (MELESSA) at the Ludwig Maximilian University Munich. An a-priori power analysis based on a meta-analysis of delay discounting in addiction reporting an effect size of Cohen’s d = 0.63^8^ suggested that 32 participants per group are sufficient to detect an effect of smoking status on delay discounting with a power of 80% (alpha = 5%). Note that such a sample size is also sufficient to reliably measure metacognitive accuracy in intertemporal decision making^24,25,28^. For study inclusion, smokers had to smoke ≥ 10 cigarettes per day and to have a score of ≥ 3 in the Fagerström Test for Cigarette Dependence^50,51^, whereas volunteers without history of regular tobacco smoking were assigned to the non-smoker group. Both smokers and non-smokers were screened for excessive alcohol consumption (inclusion criterion: AUDIT-C score ≤ 5^52^; mean_smoker_ = 3.1; mean_non-smoker_ = 2.7), other substance dependence, and neurological or psychiatric disorders. For one participant in the smoker group, data of all decision tasks (except for the risk preference task, see below) were lost due to technical issues. All participants gave voluntary informed written consent prior to participation. The study was approved by the local ethics committee of the department for psychology at Ludwig Maximilian University Munich (protocol number: 44_Soutschek_b) and performed in accordance with the Declaration of Helsinki. Study participation was re-imbursed with 10 euro/hour in addition to a performance-dependent bonus (see below).

### Stimuli and task design

*Confidence accuracy task* To assess participants’ metacognitive awareness of their time preferences, participants performed a monetary intertemporal choice task^24^. Participants chose between an immediately available smaller-sooner (SS) reward (0–5 euro today, in steps of 0.5 euro) and a larger-later (LL) reward (5 euro delivered after a delay of 1–360 days). The SS and LL reward options were randomly presented on the left or right side of the screen, and participants chose the left or right option by pressing the left or right arrow key, respectively, on a standard keyboard within 6 s. Following each choice, participants indicated their confidence that they made the best possible choice on a rating scale from 0 (low confidence) to 20 (high confidence) within 3 s (Fig. 1A). Participants made a total of 66 choices in this task, including all combinations of SS reward magnitudes (11 levels) and delays (6 levels). We note that higher LL reward magnitudes than employed in this study might increase patience due to the so-called magnitude effect^53^, which might lead to ceiling effects and reduce the sensitivity of our task to measuring individual differences in time preferences.

*Bidding task* In the bidding task^28,54^, participants indicated on a rating scale which amount of money delivered today (in steps of 0.2 euro) they considered as equivalent to 5 euro at different points of time in the future (e.g., “Which amount of money available today is equivalent to 5 euro in 90 days?”). We used the same delays as in the confidence accuracy task. This allowed us to compute the difference between the self-reported (bidding task) and the observed subjective values (based on the choices in the decision task) for each delay (Fig. 2A).

*Precommitment task*. As for the confidence accuracy task, participants made choices between SS and LL rewards. One option consisted of a fixed monetary reward of 5 euro delivered after delays of 29–388 days (precommitment option; e.g., “5 euro in 68 days”; Fig. 3A). When choosing this option, participants received 5 euro after the given delay without having the possibility to reverse their choice. The other option (postpone option) entailed an SS reward of 0–5 euro delivered after 28 days and an LL reward that was identical to the precommitment option (e.g., “3 euro in 28 days” or “5 euro in 68 days”). If for the bonus payment a trial of the precommitment task was selected where a participant had decided to postpone the final decision, the participant was re-contacted by the experimenter via email after 28 days and was asked to make a choice between the SS and LL rewards of the chosen trial, with the delays adjusted for the 28 days that had passed (in the current example, “3 euro today” or “5 euro in 40 days”). If a participant had selected the precommitment option in the chosen trial, the participant received information about the chosen option via email after 28 days without the possibility to reverse the choice. Thus, only the postpone option allowed participants to reverse their preference after 28 days. Participants performed a total of 36 choices in the precommitment task. We used 6 levels of SS reward magnitudes (2, 2.5, 3, 3.5, 4, and 4.5 euro) that were delivered after 28 days, whereas the LL reward magnitude was fixed to 5 euro and was delivered after one of 6 possible delays (29, 33, 38, 48, 68, and 118 days). These reward magnitudes and delays were adopted from our previous studies on the link between metacognitive accuracy and precommitment^24,25^.

### Procedure

After having checked participants’ eligibility, we administered a task battery including baseline measures for reward sensitivity (Behavioral Inhibition System/Behavioral Approach System (BIS/BAS) questionnaire^55^), the multiple choice vocabulary test MWT-B as proxy for verbal intelligence^56^, and working memory capacity (digit span backward task^57^). In the computerized version of the digit span backward task, participants had to report a stream of digits presented on the screen (with 3 to 9 digits) in reversed order, with each difficulty level being presented only once. For the BIS/BAS questionnaire, we computed separate scores for the BIS and BAS subscales via the subscale-specific mean rating scores (range: 1–4)^58^. These measures served to control for potential baseline differences between the smokers and non-smokers. Participants then performed the confidence accuracy task, the bidding task, and the precommitment task. Additionally, participants also performed (in counterbalanced order) decision tasks eliciting risk preferences^59,60^ and the willingness to engage in rewarded mental effort^61,62^. Because neither effort nor risk preferences have been linked to precommitment so far, the results of the latter two tasks will be reported in a separate article focusing on how smokers trade off benefits against the costs of actions. We informed participants that at the end of the experiment one of the choices made in the decision tasks would randomly be selected and the corresponding payoffs added to their payment. This established procedure ensures that participants had to take all choices seriously (as all choices were equally likely to be selected after the experiment) and avoids satisficing effects that might occur if all choices are paid out.

### Data analysis

We analyzed data with Bayesian mixed generalized linear models (MGLMs) as implemented in the brms package in R^63^. Trials with reaction times faster than 250 ms were excluded from the analyses. We also removed trials where participants failed to respond within the time limits (1% of all trials in the confidence accuracy task and 3% of all trials in the precommitment task). In the confidence accuracy task, we measured participants’ metacognitive awareness of their decision uncertainty following a previously described approach^24,25,64^. For that purpose, we first estimated each individual’s time preferences by fitting a hyperbolic discount function^65^ to the choices in the confidence accuracy task (Eq. 1):

1 $$SV_{LL} = \frac{{{\text{LL}}\;{\text{reward}}\;{\text{magnitude}}}}{{1 + k \times {\text{delay}}}}$$

where SV_LL_ is the discounted subjective value of the LL reward and k is a participant-specific constant that indicates the steepness of the discount function (“discount factor”). To translate subjective value into binary choices, we fitted a standard softmax function to each participant’s choices:

2 $$P\left( {{\text{choice}}\;{\text{of}}\;{\text{LL}}\;{\text{option}}} \right) = \frac{1}{{1 + e^{{ - \beta_{{{\text{temp}}}} \times \left( {SV_{LL} - SV_{SS} } \right)}} }}$$

This function captures the likelihood of choosing the LL reward option as a function of the difference between the subjective value of the LL reward option (SV_LL_) and the SS reward option (SV_SS_). The inverse temperature parameter β_temp_ captures the slope of the function, i.e., how strongly participants relied on this value difference for their choices. We compared the group-level estimates of the discount factor k and the inverse temperature β_temp_ by estimating group-level parameters for the smoker and non-smoker groups with a hierarchical Bayesian approach (2 chains with 4,000 samples, the first 2000 samples were used as burn-in) using the function “dd_hyperbolic” in the hBayesDM package^66^. To measure metacognitive access to individual time preferences, we computed the difference between the value of the SS reward and the subjective value of the LL reward (SV_LL_–SV_SS_) based on the individual discount factors. We then performed a Bayesian MGLM regressing binary choices (1 = LL reward, 0 = SS reward) on fixed-effects predictors for Group (0 = non-smoker, 1 = smoker), subjective value difference (DV = SV_LL_ − SV_SS_), confidence ratings, as well as all interaction terms. As random effects, we included participant-specific intercepts as well as random slopes for DV, confidence, and the interaction term. The interaction between value difference and confidence indicates the degree to which participants are aware of objective decision uncertainty in the choice process and thus constitutes a measure of metacognitive accuracy: the stronger the relationship between the slope of DV and confidence ratings, the more reliably an individual is able to track decision uncertainty in intertemporal decisions. Assessing the impact of smoking status on this interaction effect thus allowed us to test whether smokers and non-smokers differ in the metacognitive awareness of their time preferences. As in all other MGLMs, we estimated posterior distributions of parameters with two chains, each entailing 4000 samples (burn-in = 2000 samples).

In addition, we analyzed the differences between participants’ self-reported subjective values of delayed rewards in the bidding task and the subjective values as given by the individual hyperbolic discount functions derived from the decision task (see above). A positive difference between self-reported and revealed subjective reward values indicates that an individual overestimates the value of future over immediate rewards relative to their revealed preferences, whereas a negative difference indicates an underestimation of the value of delayed rewards. We regressed differences between self-reported and revealed subjective values on fixed-effect predictors for Group, Delay, and the interaction term. As random effects, we modelled participant-specific intercepts and slopes for Delay.

In the precommitment task, we asked whether smokers and non-smokers differ regarding their sensitivity to the expected benefit from precommitment. We conducted an MGLM that regressed choices in the precommitment task (0 = postpone option, 1 = precommitment option) on fixed-effect predictors for Group, Reversal risk, and value difference between SS and LL reward (DV). As random effects, we added participant-specific intercepts and random slopes for DV, reversal risk, and the interaction term. The predictor DV controls for the possibility that participants decided for the postpone option because at time of choice they preferred the SS over the LL option. We computed the reversal risk on each trial by subtracting the value difference between SS and LL reward in the perspective of 28 days later (i.e., when participants had to make a final choice between the options) from the value difference between these options in participants’ current perspective (DV) based on the individual discount factors estimated in the confidence accuracy task. A higher score indicates a higher risk of preference reversals (i.e., that a participant prefers the LL reward in the experimental session and the SS reward in 28 days) and thus a higher expected benefit from precommitting to the LL reward.

## Data Availability

The data that support the findings of this study will be available on Open Science Framework (https://osf.io/pjen2/).

## References

[1] Health, U. D. O. & Services, H. (Atlanta, GA: US Department of Health and Human Services, Centers for Disease …, 2014).

[2] Sheffer CE (2014). Delay discounting rates: A strong prognostic indicator of smoking relapse. Addict. Behav..

[3] Yoon JH (2007). Delay discounting predicts postpartum relapse to cigarette smoking among pregnant women. Exp. Clin. Psychopharm..

[4] Muraven M (2010). Practicing self-control lowers the risk of smoking lapse. Psychol. Addict. Behav..

[5] Alboksmaty A, Agaku IT, Odani S, Filippidis FT (2019). Prevalence and determinants of cigarette smoking relapse among US adult smokers: A longitudinal study. BMJ Open.

[6] Callaghan L (2021). What kind of smoking identity following quitting would elevate smokers relapse risk?. Addict. Behav..

[7] Fujita K (2011). On conceptualizing self-control as more than the effortful inhibition of impulses. Personal. Soc. Psychol. Rev..

[8] MacKillop J (2011). Delayed reward discounting and addictive behavior: A meta-analysis. Psychopharmacology.

[9] Barlow P, McKee M, Reeves A, Galea G, Stuckler D (2017). Time-discounting and tobacco smoking: A systematic review and network analysis. Int. J. Epidemiol..

[10] Pfeffer D, Wigginton B, Gartner C, Morphett K (2018). Smokers' understandings of addiction to nicotine and tobacco: A systematic review and interpretive synthesis of quantitative and qualitative research. Nicotine Tob. Res..

[11] Halpern-Felsher BL, Biehl M, Kropp RY, Rubinstein ML (2004). Perceived risks and benefits of smoking: Differences among adolescents with different smoking experiences and intentions. Prev. Med..

[12] Abdullah ASM, Ho WWN (2006). What Chinese adolescents think about quitting smoking: A qualitative study. Subst. Use Misuse.

[13] Fleming SM, Lau HC (2014). How to measure metacognition. Front. Hum. Neurosci..

[14] Maniscalco B, Lau H (2012). A signal detection theoretic approach for estimating metacognitive sensitivity from confidence ratings. Conscious. Cogn..

[15] Hamonniere T, Varescon I (2018). Metacognitive beliefs in addictive behaviours: A systematic review. Addict. Behav..

[16] Spada MM, Caselli G, Nikcevic AV, Wells A (2015). Metacognition in addictive behaviors. Addict. Behav..

[17] Moeller SJ, Goldstein RZ (2014). Impaired self-awareness in human addiction: Deficient attribution of personal relevance. Trends Cogn. Sci..

[18] Lawrance L, Rubinson L (1986). Self-efficacy as a predictor of smoking-behavior in young adolescents. Addict. Behav..

[19] Hiemstra M, Otten R, de Leeuw RN, van Schayck OC, Engels RC (2011). The changing role of self-efficacy in adolescent smoking initiation. J. Adolesc. Health.

[20] Soutschek A, Tobler PN (2018). Motivation for the greater good: Neural mechanisms of overcoming costs. Curr. Opin. Behav. Sci..

[21] Bulley A, Schacter DL (2020). Deliberating trade-offs with the future. Nat. Hum. Behav..

[22] Duckworth AL, Gendler TS, Gross JJ (2014). Self-control in school-age children. Educ. Psychol..

[23] Kurth-Nelson Z, Redish AD (2012). Don'T let me do that!— Models of precommitment. Front. Neurosci..

[24] Soutschek A, Tobler PN (2020). Know your weaknesses: Sophisticated impulsiveness drives voluntary self-restrictions. J. Exp. Psychol. Learn. Mem. Cognit..

[25] Soutschek A, Moisa M, Ruff CC, Tobler PN (2021). Frontopolar theta oscillations link metacognition with prospective decision making. Nat. Commun..

[26] Becker GS, Murphy KM (1988). A theory of rational addiction. J. Polit. Econ..

[27] Gruber J, Köszegi B (2001). Is addiction “rational”? Theory and evidence. Q. J. Econ..

[28] Bulley A, Lempert KM, Conwell C, Irish M, Schacter DL (2022). Intertemporal choice reflects value comparison rather than self-control: insights from confidence judgements. Phil. Trans. R. Soc. B..

[29] Franken IH, Muris P (2006). BIS/BAS personality characteristics and college students’ substance use. Personal. Individ. Differ..

[30] Audrain-McGovern J (2009). Does delay discounting play an etiological role in smoking or is it a consequence of smoking?. Drug Alcohol Depend..

[31] Jimura K, Chushak MS, Westbrook A, Braver TS (2018). Intertemporal decision-making involves prefrontal control mechanisms associated with working memory. Cereb. Cortex.

[32] Neighbors C, Tomkins MM, Lembo Riggs J, Angosta J, Weinstein AP (2019). Cognitive factors and addiction. Curr. Opin. Psychol..

[33] Berridge KC, Robinson TE (2016). Liking, wanting, and the incentive-sensitization theory of addiction. Am. Psychol..

[34] Bickel WK, Koffarnus MN, Moody L, Wilson AG (2014). The behavioral- and neuro-economic process of temporal discounting: A candidate behavioral marker of addiction. Neuropharmacology.

[35] Bickel WK, Marsch LA (2001). Toward a behavioral economic understanding of drug dependence: Delay discounting processes. Addiction.

[36] Krystal JH, Cramer JA, Krol WF, Kirk GF, Rosenheck RA (2001). Naltrexone in the treatment of alcohol dependence. N. Engl. J. Med..

[37] Johansson BA, Berglund M, Lindgren A (2006). Efficacy of maintenance treatment with naltrexone for opioid dependence: A meta-analytical review. Addiction.

[38] Falcone M (2016). Transcranial direct current brain stimulation increases ability to resist smoking. Brain Stimul..

[39] Dunlop K, Hanlon CA, Downar J (2017). Noninvasive brain stimulation treatments for addiction and major depression. Ann. N. Y. Acad. Sci..

[40] Bryan G, Karlan D, Nelson S (2010). Commitment devices. Annu. Rev. Econ..

[41] Bell K (2015). Thwarting the diseased will: Ulysses contracts, the self and addiction. Cult. Med. Psychiatry.

[42] Standing H, Lawlor R (2019). Ulysses contracts in psychiatric care: Helping patients to protect themselves from spiralling. J. Med. Ethics.

[43] Soutschek A (2017). Binding oneself to the mast: Stimulating frontopolar cortex enhances precommitment. Soc. Cogn. Affect. Neurosci..

[44] Shekhar M, Rahnev D (2018). Distinguishing the roles of dorsolateral and anterior PFC in visual metacognition. J. Neurosci..

[45] Moritz S, Menon M, Balzan R, Woodward TS (2022). Metacognitive training for psychosis (MCT): Past, present, and future. Eur. Arch. Psychiatry Clin. Neurosci..

[46] Crockett MJ (2013). Restricting temptations: Neural mechanisms of precommitment. Neuron.

[47] Green RM, Lawyer SR (2014). Steeper delay and probability discounting of potentially real versus hypothetical cigarettes (but not money) among smokers. Behav. Proc..

[48] Wilson SJ, MacLean RR (2013). Associations between self-control and dimensions of nicotine dependence: A preliminary report. Addict. Behav..

[49] Sayette MA, Loewenstein G, Griffin KM, Black JJ (2008). Exploring the cold-to-hot empathy gap in smokers. Psychol. Sci..

[50] Heatherton TF, Kozlowski LT, Frecker RC, Fagerstrom KO (1991). The Fagerstrom test for nicotine dependence: A revision of the Fagerstrom tolerance questionnaire. Br. J. Addict..

[51] Fagerström K (2011). Determinants of tobacco use and renaming the FTND to the Fagerström test for cigarette dependence. Nicotine Tob. Res..

[52] Bush K, Kivlahan DR, McDonell MB, Fihn SD, Bradley KA (1998). The AUDIT alcohol consumption questions (AUDIT-C): An effective brief screening test for problem drinking. Ambulatory Care Quality Improvement Project (ACQUIP). Alcohol use disorders identification test. Arch. Intern. Med..

[53] Baker F, Johnson MW, Bickel WK (2003). Delay discounting in current and never-before cigarette smokers: Similarities and differences across commodity, sign, and magnitude. J. Abnorm. Psychol..

[54] Cooper N, Kable JW, Kim BK, Zauberman G (2013). Brain activity in valuation regions while thinking about the future predicts individual discount rates. J. Neurosci..

[55] Carver CS, White TL (1994). Behavioral inhibition, behavioural activation, and affective responses to impending reward and punishment: The BIS/BAS Scales. J. Pers. Soc. Psychol..

[56] Lehrl, S. Mehrfachwahl-Wortschatz-Intelligenztest: MWT-B [Multiple Choice Vocabulary Test, version B]. *Balingen, Germany: apitta* (2005).

[57] Ramsay MC, Reynolds CR (1995). Separate digits tests: A brief history, a literature review, and a reexamination of the factor structure of the Test of Memory and Learning (TOMAL). Neuropsychol. Rev..

[58] Strobel A, Beauducel A, Debener S, Brocke B (2001). Eine deutschsprachige Version des BIS/BAS-Fragebogens von Carver und White. Zeitschrift für Differentielle und Diagnostische Psychologie.

[59] Soutschek A (2020). Dopaminergic D1 receptor stimulation affects effort and risk preferences. Biol. Psychiatry.

[60] Toubia O, Johnson E, Evgeniou T, Delquie P (2013). Dynamic experiments for estimating preferences: An adaptive method of eliciting time and risk parameters. Manage. Sci..

[61] Soutschek A, Bagaini A, Hare TA, Tobler PN (2022). Reconciling psychological and neuroscientific accounts of reduced motivation in aging. Soc. Cogn. Affect. Neurosci..

[62] Soutschek A, Kang P, Ruff CC, Hare TA, Tobler PN (2018). Brain stimulation over the frontopolar cortex enhances motivation to exert effort for reward. Biol. Psychiatry.

[63] Bürkner P-C (2017). brms: An R package for Bayesian multilevel models using Stan. J. Stat. Softw..

[64] De Martino B, Fleming SM, Garrett N, Dolan RJ (2013). Confidence in value-based choice. Nat. Neurosci..

[65] Myerson J, Green L (1995). Discounting of delayed rewards—models of individual choice. J. Exp. Anal. Behav..

[66] Ahn WY, Haines N, Zhang L (2017). Revealing neurocomputational mechanisms of reinforcement learning and decision-making with the hBayesDM package. Comput. Psychiatr..

